# Insights From Computational Modeling Into the Contribution of Mechano-Calcium Feedback on the Cardiac End-Systolic Force-Length Relationship

**DOI:** 10.3389/fphys.2020.00587

**Published:** 2020-05-29

**Authors:** Megan E. Guidry, David P. Nickerson, Edmund J. Crampin, Martyn P. Nash, Denis S. Loiselle, Kenneth Tran

**Affiliations:** ^1^Auckland Bioengineering Institute, The University of Auckland, Auckland, New Zealand; ^2^Systems Biology Laboratory, School of Mathematics and Statistics, The University of Melbourne, Melbourne, VIC, Australia; ^3^ARC Centre of Excellence in Convergent Bio-Nano Science and Technology, School of Chemical and Biomedical Engineering, The University of Melbourne, Melbourne, VIC, Australia; ^4^Department of Engineering Science, The University of Auckland, Auckland, New Zealand; ^5^Department of Physiology, The University of Auckland, Auckland, New Zealand

**Keywords:** end-systolic force-length relations, cardiac calcium transient, thin filament activation, calcium-troponin binding affinity, cardiac excitation-contraction model, mechano-calcium feedback

## Abstract

In experimental studies on cardiac tissue, the end-systolic force-length relation (ESFLR) has been shown to depend on the mode of contraction: isometric or isotonic. The isometric ESFLR is derived from isometric contractions spanning a range of muscle lengths while the isotonic ESFLR is derived from shortening contractions across a range of afterloads. The ESFLR of isotonic contractions consistently lies below its isometric counterpart. Despite the passing of over a hundred years since the first insight by Otto Frank, the mechanism(s) underlying this protocol-dependent difference in the ESFLR remain incompletely explained. Here, we investigate the role of mechano-calcium feedback in accounting for the difference between these two ESFLRs. Previous studies have compared the dynamics of isotonic contractions to those of a single isometric contraction at a length that produces maximum force, without considering isometric contractions at shorter muscle lengths. We used a mathematical model of cardiac excitation-contraction to simulate isometric and force-length work-loop contractions (the latter being the 1D equivalent of the whole-heart pressure-volume loop), and compared Ca^2+^ transients produced under equivalent force conditions. We found that the duration of the simulated Ca^2+^ transient increases with decreasing sarcomere length for isometric contractions, and increases with decreasing afterload for work-loop contractions. At any given force, the Ca^2+^ transient for an isometric contraction is wider than that during a work-loop contraction. By driving simulated work-loops with wider Ca^2+^ transients generated from isometric contractions, we show that the duration of muscle shortening was prolonged, thereby shifting the work-loop ESFLR toward the isometric ESFLR. These observations are explained by an increase in the rate of binding of Ca^2+^ to troponin-C with increasing force. However, the leftward shift of the work-loop ESFLR does not superimpose on the isometric ESFLR, leading us to conclude that while mechano-calcium feedback does indeed contribute to the difference between the two ESFLRs, it does not completely account for it.

## Introduction

The end-systolic force-length (or pressure-volume) relationships (ESFLRs) occupy a special place in cardiovascular physiology, often used as a proxy for cardiac contractility. Two distinct relationships are commonly reported, one for isometric (or isovolumic) contractions and a separate one for isotonic (or ejecting) contractions. It is sobering to reflect that the existence of these two distinct ESFLRs has been recognized since the 1899 publication by Otto Frank (1899) but remains incompletely explained. Frank’s findings arose from experiments performed on the isolated heart of the frog and clearly reveal the existence of two distinct protocol-dependent end-systolic relations. Despite Frank’s insight, and more than 100 years of research since, the mechanisms responsible for the protocol-dependence of the ESFLRs remain incompletely understood. This absence of a definitive explanation stands in stark contrast to the many experimental affirmations of the phenomenon, commencing with Brady (1967) and Gibbs et al. (1967), who independently demonstrated that the isometric ESFLR of isolated papillary muscles lies to the left of the equivalent isotonic (or work-loop) curve.

This phenomenon can be readily replicated and a variety of explanations have been proffered. These include the contribution of strain rate-dependent transitions between strong and weak cross-bridge conformations (Landesberg, 1996), velocity-dependent shortening deactivation of thin filaments (Iribe et al., 2014), and sarcomeric force-length dynamic interactions (Pironet et al., 2013). Perhaps the most germane of which was that of Brady (1967): “the time-course of the active state,” which focused attention on the duration of the Ca^2+^ transient. But it was Housmans et al. (1983) who demonstrated that active shortening retards the decline of the Ca^2+^ transient, a phenomenon that their group boldly attributed to the affinity of Ca^2+^ for troponin-C, hinting at the role of mechano-calcium feedback. Their insight has been corroborated by many others, in both intact (ter Keurs et al., 1980; Lab et al., 1984; Kentish and Wrzosek, 1998) and skinned (Hibberd and Jewell, 1982) cardiac muscle preparations, and in both relaxed and rigor states, thereby demonstrating the requirement that cross-bridges be attached for the phenomenon to instantiate (Hofmann and Fuchs, 1987; Kurihara et al., 1990). The encompassing phrase “myofilament length-dependent activation” includes the now widely accepted notion of force-dependent binding of Ca^2+^ to troponin (Rice and de Tombe, 2004; de Tombe et al., 2010; Solaro and Kobayashi, 2011; de Tombe and ter Keurs, 2016; Li et al., 2016), where force-dependence arises primarily from the consequence of length-dependent overlap of the thick and thin contractile filaments. At the core of this mechanism is the role of Ca^2+^ as an activator of contraction and as a target for force feedback—the mechano-calcium mechanism.

A shortcoming of previous experimental studies has been the comparison of isotonic contractions to an isometric contraction at the unique length (L_o_) that maximizes force (Housmans et al., 1983; Lab et al., 1984). While such studies have uncovered a number of key properties of actively shortening muscle, the failure to compare isotonic contractions to isometric contractions of equivalent force has hindered the development of a satisfactory explanation to explain their different ESFLRs.

This perceived shortcoming has prompted us to investigate the role of mechano-calcium feedback in the mode-dependence of the ESFLR and to ask whether the Ca^2+^ transients of isometric- and work-loop-derived ESFLRs are different under equivalent force conditions. If that were to transpire, then a second question would arise: could the activation of work-loop contractions using Ca^2+^ transients derived from isometric contractions reconcile these two ESFLRs? To that end, we undertook the assembly of a mathematical model of cardiac excitation-contraction. The model tightly couples three existing components—one for each of electrical excitation (Rogers and McCulloch, 1994), Ca^2+^ dynamics (Hinch et al., 2004) and cross-bridge mechanics (Tran et al., 2017). The latter module, which is based on a three-state model of cross-bridge cycling (Rice et al., 2008), is capable of generating both isometric and afterloaded isotonic contractions under either fixed or variable Ca^2+^ transients. It thus provides a tool with which to address the two questions raised above.

## Materials and Methods

### Model Development

A novel integrated model of cardiac cellular excitation-contraction was developed to investigate the protocol-dependence of the isometric and work-loop ESFLRs. The model was designed to encapsulate sufficient biophysical detail for the purposes of the current study, with a focus on computational efficiency to permit integration into large scale tissue modeling studies.

**FIGURE 1:**
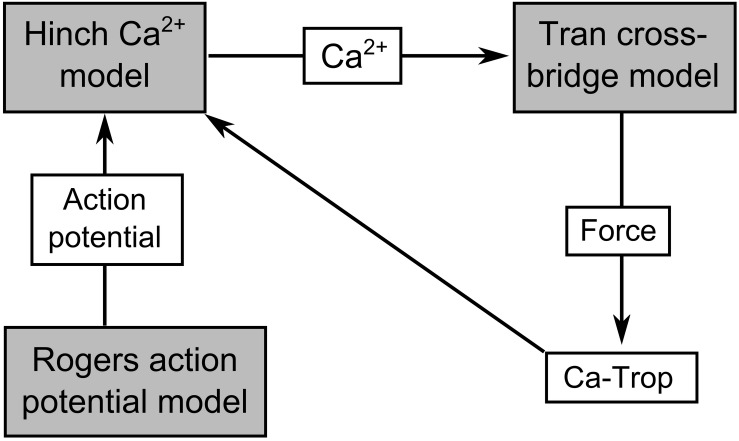
Schematic of the THR excitation-contraction model. The connections indicate the coupling between the Tran et al. (2017) cross-bridge model, the Hinch et al. (2004) Ca^2+^ model, and the Rogers and McCulloch (1994) action potential model. Ca-Trop represents Ca^2+^ bound to troponin-C, which is modulated by active force, and therefore mediates the mechano-calcium feedback pathway.

The Tran et al. (2017) mechano-energetics model forms the basis of the coupled model, since it captures the detailed biophysical interdependencies of thin filament activation and cross-bridge cycling. It has been previously parameterized to simulate a wide range of experimentally observed cardiac myofilament behavior, including steady-state, force-length, and force-Ca^2+^ relations, as well as dynamic force transients and metabolite dependence (Rice et al., 2008; Tran et al., 2010). The force-dependent binding of Ca^2+^ to troponin-C is also explicitly captured allowing cross-bridge force production to modify the intracellular Ca^2+^ transient. The total force generated by the model is composed of an active and a passive component. To enable the required range of simulations, the cross-bridge model was coupled to the Hinch et al. (2004) model of Ca^2+^-induced-Ca^2+^-release (CICR), which simulates Ca^2+^ fluxes across the sarcolemmal and sarcoplasmic reticular membranes. The CICR model was driven by a simplified model of the cardiac action potential. Since our study did not require detailed cellular electrophysiology, we parameterized the phenomenological Rogers and McCulloch (1994) model to simulate a rat cardiac action potential and used it to drive the cellular contraction cycle. The mechano-calcium feedback between the cross-bridge and CICR models was implemented using equations 56–64 of Rice et al. (2008). All three components of the model were previously parameterized for rat cardiomyocyte, and were not modified from their original values in this study. The Rogers and McCulloch (1994) action potential model is not temperature dependent, while the Hinch et al. (2004) model was parameterized using data at 22°C and the Tran et al. (2017) cross-bridge model is appropriate over the temperature range of 23–37°C. We, therefore, set the temperature of the Tran et al. (2017) cross-bridge model to 23°C to be consistent with that of Hinch et al. (2004). A schematic of the integrated Tran-Hinch-Rogers (THR) model is presented in Figure 1. The simulated calcium transient therefore arises from interactions between the dynamics of the CICR model and the mechano-calcium feedback of the cross-bridge model.

Each of the Rogers and McCulloch (1994); Hinch et al. (2004), and Tran et al. (2017) models is available in the Physiome Model Repository.^1^ We took advantage of the modular features of the CellML standard (Cuellar et al., 2003) and the software tool OpenCOR (Garny and Hunter, 2015) to integrate these disparate models to construct the coupled THR model of cardiac excitation-contraction (see Nickerson and Buist, 2008; Terkildsen et al., 2008 for detailed demonstrations of such model integration). The CellML models, descriptions of simulation experiments, and parameters for reproducing the figures in this study are available from: https://models.physiomeproject.org/workspace/4ca.

### Isometric and Work-Loop Protocols

The THR model was used to simulate isometric and work-loop contractions. Isometric simulations consisted of holding the sarcomere length constant throughout the duration of a contraction. Initial sarcomere lengths were varied between 1.95 and 2.3 μm, the length (L_o_) at which maximum force is produced. At each sarcomere length, twitches were simulated at 1 Hz frequency until a beat-to-beat steady-state was reached (20 beats). The isometric ESFLR was derived by plotting the peak force as a function of sarcomere length. All forces in the simulations were normalized to the peak L_o_-isometric force.

The work-loop protocol was designed to mimic the whole-heart pressure-volume loop (Taberner et al., 2011), and was simulated using four contraction phases.

#### Phase 1: Isometric Contraction

Starting at an initial sarcomere length of L_o_, stimulation by an action potential initiates CICR and force generation. Muscle length is held constant as force develops. Isometric contraction continues until the total muscle force (the sum of active and passive forces) exceeds a user-specified afterload.

#### Phase 2: Isotonic Contraction

Following Phase 1, once the muscle force exceeds the afterload, the muscle shortens isotonically against the constant afterload. The shortening velocity is calculated using Equation 38 from Rice et al. (2008). The simulation continues in this phase for as long as the force generated by the muscle matches or exceeds the user-specified afterload.

#### Phase 3: Isometric Relaxation

When the force generated by the muscle falls below that of the afterload, it is declared to have reached end-systole. The muscle length is held constant while the force decreases until Ca^2+^ declines to diastolic levels.

#### Phase 4: Re-stretch

When Ca^2+^ reaches diastolic levels, the now-quiescent muscle is re-stretched, at constant velocity, to its initial length of L_o_.

Work-loops were simulated using afterloads between 0.2 and 1 (normalized to isometric force at L_o_). Work-loops at a normalized afterload of 1 produced contractions that were identical to an L_o_-isometric contraction, because the afterload exceeded the threshold for muscle shortening. Similar to the isometric protocol, a train of work-loop contractions was generated to attain steady-state at a frequency of 1 Hz. The work-loop-derived ESFLR was given by the force and length of the muscle at the end of Phase 2 (end-systole) for each work-loop contraction. The sarcomere lengths for the isometric contractions and afterloads for the work-loop contractions were chosen so that they formed pairs of force-equivalent contractions. This allowed the comparison of force profiles and Ca^2+^ dynamics for the two modes of contraction at the same level of force production.

## Results

### Model Validation

**FIGURE 2:**
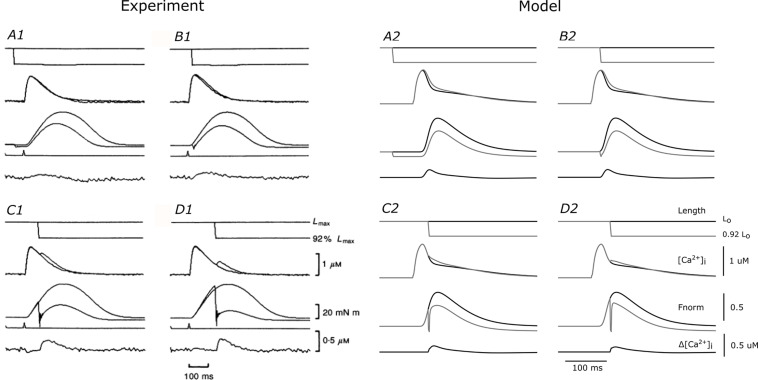
Comparison of THR model simulations with experimental quick-release data. Experimental quick-release shortening of ferret papillary muscle by Kurihara and Komukai (1995) (left) at 30°C and equivalent simulations from the THR model (right). Each of the four panels for the experimental (A1–D1) and model (A2–D2) outputs represent different timings of the quick release. Within each panel are traces of outputs from isometric (black lines) and quick-release protocols (gray lines). From top to bottom, the traces represent muscle length, intracellular Ca^2+^ concentration, total force development, and difference of Ca^2+^ between the two protocols. (A1–D1 were reproduced with permission from John Wiley and Sons).

We used the coupled THR model to examine the role of Ca^2+^ in the ESFLR of cardiac muscle contraction. The Ca^2+^ and force dynamics of the model were validated by comparing simulated output to quick-release shortening experiments reported by Kurihara and Komukai (1995). In those experiments, isolated cardiac muscles were initially held at a length (L_o_) that produced maximum force, and then was rapidly shortened to a new length (0.92 L_o_) at four different predetermined times following the stimulus. The resulting force and Ca^2+^ transients were measured and compared to those of an isometric contraction where the length was held constant at L_o_ throughout the duration of the twitch (Figure 2). The sudden length change due to the quick-release caused an immediate drop in developed force and a near-simultaneous increase of intracellular Ca^2+^, evidenced by the extra surge of Ca^2+^ during the declining phase of the Ca^2+^ transient. The magnitude of the extra Ca^2+^ elicited by the quick-release twitch is shown in the lower-most trace in each panel of Figure 2.

We simulated the quick-release protocol in our THR model and semi-quantitatively reproduced the experimental data from Kurihara and Komukai (1995). Note that none of the parameters in the THR model were optimized to reproduce the experimental data. The quick release from L_o_ to 0.92 L_o_ caused a decrease in force and an increase in intracellular Ca^2+^. The rates of force redevelopment in the model simulations were greater than those of the experiments, as is evident in panels C1 and D1 (experiment) versus C2 and D2 (model) of Figure 2, where the length change was applied after the peak of the Ca^2+^ transient. This may be due to the inherently faster cross-bridge cycling rates of the rat (model) versus those of other larger mammalian species, such as the ferret (Hasenfuss et al., 1991; Palmer and Kentish, 1998). The Ca^2+^ surges in panels C2 and D2 were smaller than those recorded in the experiments (panels C1 and D1), because the recovery force was much larger. Nevertheless, the force recovery and Ca^2+^ surge were semi-quantitatively similar to the experimental data in panels A1 and B1, thereby validating the ability of the THR model to mimic force-dependent binding of Ca^2+^ from troponin-C and the consequent effect on the simulated Ca^2+^ transient.

### Simulating Isometric and Work-Loop ESFLRs Using Dynamic Ca^2+^

**FIGURE 3:**
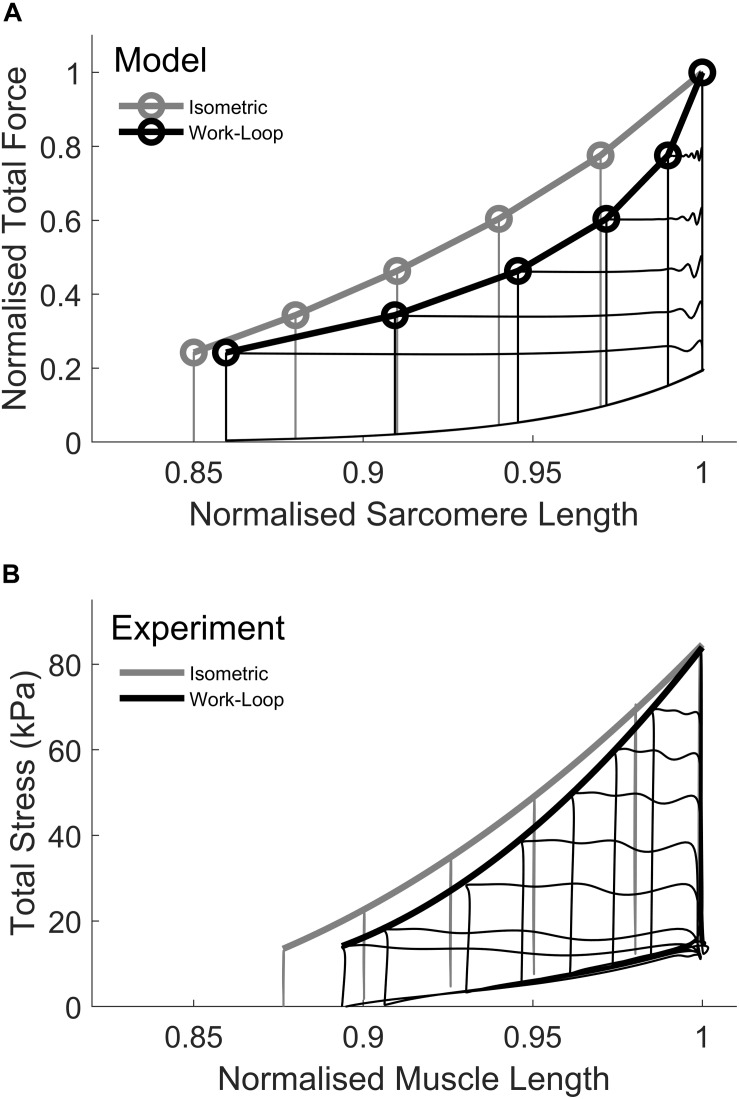
Simulation of isometric and work-loop end-systolic force-length relations (ESFLR). **(A)** The isometric ESFLR (gray) was simulated from six isometric contractions at sarcomere lengths ranging from 1.95 to 2.3 μm (L_o_). Sarcomere lengths were normalized to L_o_. The work-loop ESFLR was simulated from five work-loop contractions commencing at an initial sarcomere length of L_o_. Each work-loop contraction is matched by an isometric contraction of equivalent force. Note that the work-loop contraction at the highest afterload is identical to the L_o_-isometric contraction. **(B)** Experimental data depicting the isometric ESFLR and work-loop ESLFR from left ventricular rat trabeculae. Reproduced from data reported in Tran et al. (2016).

The ESFLRs for isometric and work-loop contractions were simulated using the THR model following the protocols outlined in Section “Materials and Methods” (Figure 3A). The isometric ESFLR was derived from isometric contractions at different sarcomere lengths while the work-loop ESFLR was derived from afterloaded shortening contractions, all of which commenced at the same initial length (L_o_). These simulation results compare well with the experimental data from rat trabeculae (Tran et al., 2016) in Figure 3B and are consistent with other previous studies on rat cardiac myocytes (Iribe et al., 2014), rat trabeculae (Han et al., 2014a, b), rabbit papillary (Sørhus et al., 2000), ferret papillary (Hisano and CooperIV, 1987), and rat papillary (Gülch, 1986), the simulated work-loop ESFLR lies to the right of its isometric counterpart. In Figure 3A, the afterloads for the simulated work-loop contractions (black circles) correspond to isometric contractions of equivalent peak force (gray circles). For a given afterload, a work-loop contraction did not shorten to the sarcomere length of its force-equivalent isometric contraction. Previous studies have attributed this difference to mechano-calcium feedback, a phenomenon whereby active shortening of a muscle during a contraction reduces the affinity of Ca^2+^ for troponin-C, thus offloading Ca^2+^ into the cytosol and reducing the level of activation of the cross-bridges (Housmans et al., 1983; Lab et al., 1984; Baan et al., 1992). The principal mechanism for this phenomenon is attributed to the force-dependent binding of Ca^2+^ to the troponin-C complex, whereby the affinity of Ca^2+^ for troponin-C increases with increasing force (Hibberd and Jewell, 1982; Hofmann and Fuchs, 1987). For our simulated work-loop contractions, shortening during the isotonic phase (Phase 2) is expected to offload Ca^2+^ from troponin-C and enhance the descending portion of the intracellular Ca^2+^ transient. The level of enhancement is also predicted to increase with decreasing afterload.

**FIGURE 4:**
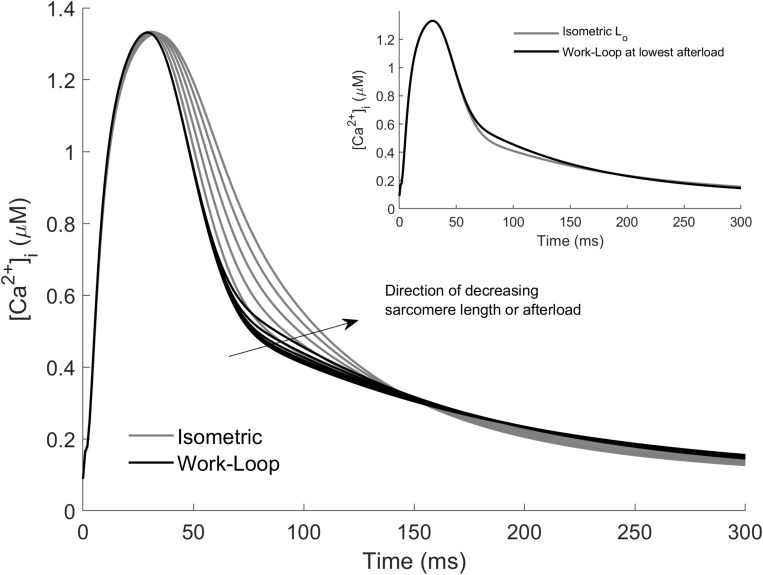
Simulated Ca^2+^ transients arising from isometric and work-loop contractions. Isometric contractions (gray) as functions of initial sarcomere length and work-loop contractions (black) as functions of afterload. The arrow indicates the direction of decreasing sarcomere length for the isometric simulations, and decreasing afterload for the work-loop simulations. Inset: Ca^2+^ transients of the L_o_-isometric contraction (gray) and the work-loop contraction (black) at the lowest afterload.

Model simulations of the Ca^2+^ transients associated with work-loop contractions are presented in Figure 4. The work-loop contractions (black lines) did indeed prolong the Ca^2+^ transient compared to the isometric contraction at L_o_. The Ca^2+^ transients widened with decreasing afterload, but the extent of widening was small relative to the effect of decreasing sarcomere length on the isometric contractions (Figure 4, gray lines). The narrowest of the isometric Ca^2+^ transients corresponds to an isometric contraction at L_o_ (gray line in Figure 4 inset). Note that because all of the work-loop contractions originated at L_o_, the Ca^2+^ transients for the work-loop contractions matched those of the L_o_-isometric contraction up to the onset of Phase 2 (i.e., up to *t* = 45 ms). Decreasing the muscle length of the isometric contractions resulted in a more significant widening of the Ca^2+^ profile relative to the effect of decreasing afterload in the work-loop contractions. For clarity, the Ca^2+^ transients for the L_o_-isometric contraction and the work-loop contraction at the lowest afterload are shown in the inset of Figure 4. Additional simulations of work-loop ESFLRs at initial sarcomere lengths below L_o_ were consistent with recent experimental data on rat cardiac trabeculae showing the preload dependence of work-loop contractions (Han et al., 2019), and simulation of associated Ca^2+^ transients were consistent with the trends observed in Figure 4 (Supplementary Material).

**FIGURE 5:**
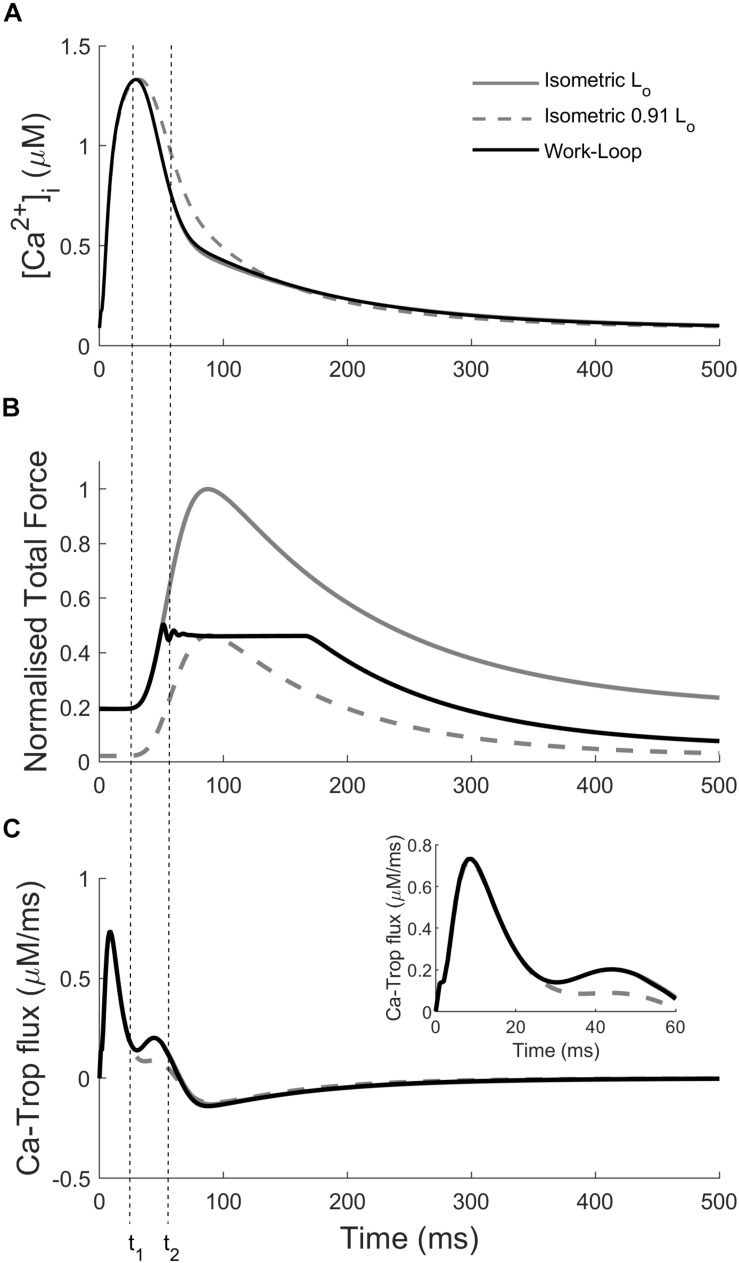
Simulations highlighting the mechano-calcium feedback mechanism. Simulated intracellular Ca^2+^ transient **(A)**, total force production **(B)**, and Ca-troponin flux **(C)** for an L_o_-isometric contraction, and a pair of isometric (SL = 0.91 L_o_) and work-loop (normalized afterload at 0.45) contractions at the same peak force. Inset: close-up of the Ca-troponin flux at the start of the twitch. The dotted vertical lines indicate the times at the start of force production (t_1_) and at the start of the isotonic phase in the work-loop contraction (t_2_).

The variation of the Ca^2+^ transients within and between the two modes of contraction (Figure 4) is a result of the interaction between cross-bridge force production and the force-dependent affinity of Ca^2+^ for troponin-C. In Figure 5, the intracellular Ca^2+^ concentration, force, and the flux of Ca^2+^ binding to troponin (Ca-troponin) as functions of time are presented for the L_o_-isometric contraction, as well as for a selected pair of isometric and work-loop contractions at equivalent peak forces. The force-equivalent isometric contraction was simulated at SL = 0.91 L_o_, while the force-equivalent work-loop was simulated at a normalized afterload of approximately 0.45. The narrowest of the three Ca^2+^ transients was generated by the L_o_-isometric contraction, while the widest was generated by the 0.91 L_o_-isometric contraction. The variations in the Ca^2+^ profiles were directly due to the differential responses of the Ca-troponin fluxes to the contractions.

The Ca-troponin fluxes began diverging at *t* = 20 ms when the active force in each of the three contractions started to rise (Figure 5; *t*_1_). The faster rate of force development in the L_o_-isometric and work-loop contractions transiently increased the Ca-troponin flux above that of the 0.91 L_o_-isometric contraction, which had a lower rate of force development over that initial period. As a result, less Ca^2+^ was bound to troponin for the 0.91 L_o_-isometric contraction, which was reflected in the widening of its Ca^2+^ transient. Intracellular Ca^2+^ concentration directly affects the level of cross-bridge activation and the ability of the muscle to maintain force. For given equivalent peak forces, the model simulations reveal that isometric contractions were characterized by wider Ca^2+^ transients, relative to those of work-loop contractions (Figure 4). We hypothesized that driving work-loop contractions with wider Ca^2+^ transients would result in greater isotonic shortening and a leftward shift of the work-loop derived ESFLR toward the isometric ESFLR.

### Simulating Work-Loop ESFLRs Using Fixed Ca^2+^ Transients

**FIGURE 6:**
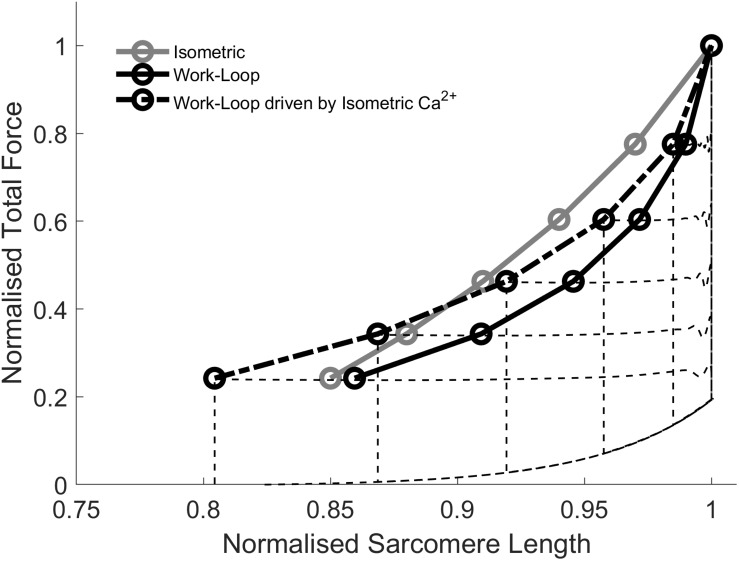
Simulations of work-loops driven by Ca^2+^ transients generated from isometric contractions. The isometric and work-loop ESFLR are replotted from Figure 4. Driving work-loops with Ca^2+^ transients generated from force-equivalent isometric contractions leads to a left-ward shift of the work-loop end-systolic force-length relation (ESFLR; dashed line) but it does not superimpose on the isometric ESFLR.

Figure 6 shows simulation results whereby Ca^2+^ transients associated with isometric contractions (Figure 4) were used to drive force-equivalent work-loop contractions. The activation of work-loop contractions using the wider Ca^2+^ transients derived from isometric contractions caused a leftward-shift of the work-loop derived ESFLRs (Figure 6). Specifically, the greater level of activation increased the proportion of cross-bridges in force-producing states, leading to greater force production at lower sarcomere lengths.

The extent of the shift increased with decreasing afterloads. At high afterloads, the wider Ca^2+^ transients were not sufficient to shift the work-loop end-systolic point to coincide with its isometric counterpart. However, at afterloads below approximately 0.4 normalized total force, the work-loop end-systolic point was shifted beyond its isometric counterpart. The two ESFLRs therefore crossed over at around the midpoint. These simulations demonstrate that the work-loop derived ESFLR can be shifted toward the isometric ESFLR by increasing the duration of the Ca^2+^ transient to prolong the shortening phase. But the contraction-mode difference in the Ca^2+^ transients cannot completely explain the discrepancy between the isometric and work-loop ESFLRs.

## Discussion

Using computational modeling, we have demonstrated that mechano-calcium feedback in cardiac muscle produces Ca^2+^ transients, which are dependent on the mode of contraction. We compared the force and Ca^2+^ dynamics of work-loop contractions with their force-equivalent isometric counterparts (Figure 5) and revealed that the Ca^2+^ transients generated by isometric contractions were of longer duration (Figure 4). Our computational modeling approach enabled the application of a novel “cross-over” technique wherein work-loops are driven with Ca^2+^ transients generated by force-equivalent isometric contractions (Figure 6), a feat which has yet to be realized experimentally. However, the difference in the duration of Ca^2+^ transients is insufficient to completely explain the protocol-dependent difference between the isometric- and work-loop-derived ESFLRs. At higher afterloads, the increase in the Ca^2+^ transient duration was insufficient to overcome the shortening deactivation that is present in the cross-bridge model (Rice et al., 2008). Specifically, muscle shortening reduces cross-bridge distortion, which promotes cross-bridge detachment and leads to a loss of activation and a reduction in tension. At lower afterloads, the Ca^2+^ transients generated by force-equivalent isometric contractions were longer, overcompensating for the shortening deactivation, resulting in a shift of the work-loop end-systolic points past those generated from isometric contractions.

The mechanism of force-dependent affinity of Ca^2+^ to troponin-C is responsible for the prolongation of the Ca^2+^ transient duration for both modes of contraction (Figure 5). Model simulations revealed that reducing the initial muscle length of an isometric contraction produced a much more significant widening of the Ca^2+^ transient than decreasing the afterload for a work-loop contraction. Isometric contractions therefore operate at a greater level of Ca^2+^ activation, explaining the leftward-shift of the isometric ESFLR. Our conclusion is in accord with the study of De Tombe and Little (1994) on rat cardiac trabeculae at 27°C, which showed that the ESFLR of shortening contractions is left-shifted with respect to isosarcometric contractions when the duration of activation is prolonged by substitution of Ca^2+^ with Sr^2+^. Our simulations reveal that the difference in the ESFLR of these two modes of contraction cannot be solely explained by the differing levels of activation elicited through the mechanism of force-dependent affinity of Ca^2+^ binding to troponin-C.

### Force-Equivalent Contractions

Previous experimental studies investigating the protocol-dependence of the ESFLR compared isotonic contractions to a single isometric contraction at L_o_ (Housmans et al., 1983; Lab et al., 1984). Housmans et al. (1983) initially hypothesized that: “The rightward location of work-loop end-systolic points is due to sarcomere shortening abbreviating the Ca^2+^ transient responsible for excitation-contraction coupling” but rejected this hypothesis when their experimental data showed that the isotonic contraction actually exhibited a wider Ca^2+^ transient than the isometric case. Instead, they proffered the hypothesis that the “extra” Ca^2+^ released during the relaxation phase of the Ca^2+^ transient appears too late in the twitch cycle to have an effect on prolongation of force development.

Our simulation results are consistent with these two studies provided that we replicate their protocol by comparing the Ca^2+^ transient from an afterloaded work-loop contraction to that of a single isometric contraction at L_o_ (Figure 4 inset). The Ca^2+^ transient of the work-loop contraction was indeed wider than that of the L_o_-isometric contraction. Compared to the experimental results of Housmans et al. (1983) and Lab et al. (1984), the magnitude of the increase of Ca^2+^ in our simulations was less pronounced. This is likely to be because the lowest afterload of our work-loop simulations is not as low as those achieved in the experiments owing to the lower passive force component in experimental preparations relative to that of our model. But this reveals only part of the story, since we showed that the greater prolongation of the Ca^2+^ transient for a work-loop contraction holds only when compared to the L_o_-isometric contraction. For isometric contractions at reduced muscle lengths, the predicted Ca^2+^ transient is of a longer duration than that of the work-loop contractions (Figure 4). In other words, a work-loop *does* have an abbreviated Ca^2+^ transient when compared to an isometric contraction that is of equivalent peak force (Figure 5).

### Time-Course of Isometric and Work-Loop Twitches

The end-systolic point of an isometric contraction coincides with the peak force of the twitch, whereas the end-systolic point of a work-loop contraction is defined as the point where the muscle can no longer shorten. As a result, the end-systolic point of a work-loop contraction always occurs later in the time-course of the Ca^2+^ transient when its intracellular Ca^2+^ concentration is lower (Figure 5). Therefore, the width of the Ca^2+^ transient, governed by the rate at which it declines, is a key factor that determines the positioning of the work-loop derived ESFLR. By widening the Ca^2+^ transient, more time is available for shortening to occur before Ca^2+^ levels fall too low (Figure 6).

The different time-courses of isometric and work-loop contractions also explain their varying effects on the width of the Ca^2+^ transient. Decreasing the afterload of a work-loop contraction has a relatively small impact on the Ca^2+^ transient compared to decreasing the initial length of an isometric contraction (Figure 4). Every work-loop starts Phase 1 at an initial length of L_o_ and behaves like an L_o_-isometric contraction during Phase 1. The mechanical feedback of isotonic shortening onto the affinity of Ca^2+^ for troponin-C does not occur until Phase 2, which is approximately 45 ms after the start of the Ca^2+^ transient (Figure 5; t_2_). This time point is well into the declining phase of the Ca^2+^ transient; hence, the enhancement of the transient from this point on is relatively small. In contrast, for an isometric contraction, the muscle length is changed prior to stimulation of the muscle. The commencement of the twitch takes place 25 ms after the start of the Ca^2+^ transient (Figure 5; t_1_). Hence, for isometric contractions at reduced initial muscle lengths, the mechanical feedback begins to affect the Ca^2+^ transient from an earlier time point, leading to a greater increase in the width of the Ca^2+^ transient relative to decreasing the afterload in work-loop contractions. Mechanical feedback from isometric contractions arises from the positive relationship between muscle length and the initial rate of tension development. At decreasing muscle lengths, the rate of rise of tension development also decreases (Han et al., 2014a, b; Tran et al., 2016), which lowers the Ca-troponin flux, allowing enhancement of the entire declining phase of the Ca^2+^ transient (Figure 5).

### Time-Course of Ca^2+^ Transients

The duration of our simulated Ca^2+^ transients increased with decreasing sarcomere length and decreasing afterload for isometric and work-loop contractions, respectively (Figure 4). The prolongation of the Ca^2+^ transient associated with isometric contractions at shorter muscle lengths is consistent with experimental studies on isolated rat cardiac tissues (Allen and Kurihara, 1982; Kentish and Wrzosek, 1998). At shorter muscle lengths, the affinity of Ca^2+^ for troponin-C is lower, hence less Ca^2+^ is bound to troponin-C, resulting in a wider Ca^2+^ transient. The effect of an afterloaded contraction on the Ca^2+^ transient has typically been compared only to an L_o_-isometric contraction, as has been discussed above.

Despite a prolongation of the Ca^2+^ transient due to either a reduction of initial muscle length in isometric contractions or a reduction of afterload in work-loop contractions, the peaks of the simulated Ca^2+^ transients remained relatively constant for both modes of contraction. Studies on rat cardiac trabeculae have reported no change in the peak Ca^2+^ transient in isometric contractions at different muscle lengths (Allen and Kurihara, 1982; Backx and Ter Keurs, 1993; Kentish and Wrzosek, 1998). Studies on the effect of a shortening contraction on the peak of the Ca^2+^ transient are not so clear. Peak Ca^2+^ has been shown to be higher for a shortening contraction compared to an isometric contraction in rat cardiac myocytes (Yasuda et al., 2003) and trabeculae (Janssen and de Tombe, 1997). In contrast, no differences were reported for either guinea pig myocytes (White et al., 1995), or for papillary muscles of either ferret (Lab et al., 1984) or cat (Housmans et al., 1983). In rat cardiomyocytes undergoing auxotonic contractions, Hongo et al. (1996) showed no change in the Ca^2+^ transient peak immediately after a stretch, but a gradual increase in peak force and peak Ca^2+^ transient 3 min later.

In our simulations, the peak of the Ca^2+^ transient remained relatively constant, because only a fraction of peak force development had occurred at the time of peak Ca^2+^ (Figure 5). The completion of force development occurs after the peak Ca^2+^ has passed and is reflected in the prolongation of the declining phase of the Ca^2+^ transient (Allen and Kurihara, 1982).

### Comparison to Previous Computational Modeling Studies

A number of studies have developed computational approaches to investigate the ESFLR of cardiomyocytes (Iribe et al., 2014) and the end-systolic pressure-volume relation (ESPVR) of the whole heart (Landesberg and Sideman, 1994; Landesberg, 1996; Shimizu et al., 2002). Iribe et al. (2014) were able to reconcile their protocol-dependent ESFLR data collected from rat cardiomyocytes by modifying an excitation-contraction model to include a term that represents the shortening velocity-dependent inactivation of the thin filament. However, despite the absence of this mechanism in our THR model, we were still able to produce the contraction-mode dependence of the ESFLR, suggesting that velocity-dependent inactivation of thin filaments is not the sole mechanism. In our THR model, the protocol-dependence of the ESFLR was attributed to force-dependent affinity of Ca^2+^ to troponin-C, a mechanism that is in accord with that of many other modeling studies that describe thin filament activation and cross-bridge force generation (Katsnelson et al., 1990; Izakov et al., 1991; Markhasin et al., 2003; Niederer et al., 2006; Syomin and Tsaturyan, 2017).

Our model simulations of the mode-dependency of the ESFLR (Figure 3) and the effect of changing initial sarcomere length on the Ca^2+^ transient from isometric contractions (Figure 4) are consistent with the model simulations of Landesberg and Sideman (1994), which incorporated force-dependent affinity of troponin-C for Ca^2+^ as well as velocity-dependent transitions between weak and strong cross-bridge states in their four-state model of cross-bridge contraction. In subsequent work, Landesberg (1996) showed that the ESPVR of work-loop contractions could be shifted by varying the scale of the velocity-dependence parameter. However, in that model, the effect of changing initial sarcomere length on the Ca^2+^ transient from isometric contractions was reversed, i.e., longer initial sarcomere lengths resulted in wider, rather than narrower, Ca^2+^ transients. We are unable to determine why the behavior of the Ca^2+^ transient is inconsistent between those two publications by Landesberg except to point out that in Landesberg (1996), a simple thin-wall model of the LV was added.

In experiments on canine hearts, Shimizu et al. (2002) reported a single ESPVR for isovolumic and ejection contractions and invariant shape of the Ca^2+^ transient for both modes of contraction. As such, they used a single Ca^2+^ transient to drive their model of cross-bridge force development. They concluded that length-dependence of the Ca^2+^ binding affinity for troponin-C was not an important factor in the canine heart, which is at odds with our findings using the THR model of rat myocardium. This disparity is likely due to the peculiarity of canine hearts, which have been reported to exhibit an ESPVR that is independent of contraction protocol (Taylor et al., 1969; Suga and Sagawa, 1974; Weber et al., 1976; Suga et al., 1981). Contraction-mode independence of the ESPVR is likely an artifact arising from performance of work-loop contractions at low diastolic left ventricular volumes (Han et al., 2019). These findings stand in contrast to those from the hearts of other smaller mammalian species (ferret, rabbit, and rat) (Hisano and CooperIV, 1987; Sørhus et al., 2000; Han et al., 2014a, b; Tran et al., 2016), all of which show contraction-mode dependence.

### Model Limitations

The THR model of excitation-contraction utilizes a simple phenomenological model of rat cardiac electrophysiology to capture the action potential waveform, and couples it to detailed biophysical models of Ca^2+^ handling and cross-bridge kinetics. The phenomenological nature of the action potential waveform means that electrophysiological length-dependent pathways, such as stretch-activated channels (SACs) which underlie the slow force response (Calaghan and White, 2004; Niederer and Smith, 2007; Dowrick et al., 2019), are not explicitly captured. But despite their absence, the THR model is able to reproduce the widely observed difference in isometric and work-loop ESFLRs, suggesting that length-dependent electrophysiological pathways play a minor role, if any, in eliciting this phenomenon.

The contraction-mode dependence of the cardiac ESFLR has been observed to be independent of experimental temperature (Gülch, 1986; Hisano and CooperIV, 1987; Sørhus et al., 2000; Han et al., 2014a, b; Tran et al., 2016), providing confidence in the predictions of our model, which was parameterized using data at room temperature. Experimental reports from cat papillary (Downing and Sonnenblick, 1964) and frog myocytes (Parikh et al., 1993) at room temperature, which purported to show contraction-mode independence of the ESFLR, was recently refuted in a re-examination of the data using a novel end-systolic zone framework (Han et al., 2019).

### Implications for Cardiac Physiology

The mechanical loading experienced by individual cardiomyocytes in the heart wall during a contraction cycle is heterogeneous. The tension-time-courses generated by cardiomyocytes vary transmurally from endocardial to the epicardial surfaces (Khokhlova et al., 2020). Mechanical feedback mechanisms allow communication of, and adaptation to, changing loading conditions across individual cells and over regions of myocardium. In the case of mechano-calcium feedback, facilitated by binding of calcium to troponin C, this mechanism allows modulation of twitch dynamics in response to changes in local loading conditions.

The impact of pathological changes on mechano-calcium feedback on cardiac function, in particular, on the ESFLR is not well known. Our recent work demonstrates that in healthy cardiac tissues, the end-systolic zone (defined as the region enclosed by isometric and work-loop ESFLRs; see Han et al., 2019) increases in the presence of isoproterenol-induced enhancement of contractility (Tran et al., 2020). We would expect the end-systolic zone to also be impacted in a disease setting. We note that mutations in the gene that encodes for cardiac troponin C (TNNC1) have been associated with hypertrophic cardiomyopathy (Liang et al., 2008; Pinto et al., 2009; Parvatiyar et al., 2012), restrictive cardiomyopathy (Parvatiyar et al., 2010), and ventricular fibrillation (Parvatiyar et al., 2012). While these pathologies are associated with the role of troponin C as an activator of contraction, changes in Ca^2+^ binding and unbinding kinetics can lead to modification of the calcium transient.

## Data Availability Statement

Publicly available datasets were analyzed in this study. These data can be found here: Kurihara and Komukai (1995). Tension-dependent changes of the intracellular Ca^2+^ transients in ferret ventricular muscles. J Physiol 489 (Pt 3), 617–625.

## Author Contributions

KT, MN, DL, and MG conceived and designed the experiments. MG and DN performed the computational simulations. All authors provided critical feedback on simulation results and manuscript revision, and all approved the final manuscript.

## Conflict of Interest

The authors declare that the research was conducted in the absence of any commercial or financial relationships that could be construed as a potential conflict of interest.

## Footnotes

1 https://models.physiomeproject.org

## References

[B1] Allen D. G. Kurihara S. (1982). The effects of muscle length on intracellular calcium transients in mammalian cardiac muscle. *J. Physiol.* 327 79–94. 10.1113/jphysiol.1982.sp014221 7120151 PMC1225098

[B2] Baan J. van der Velde E. T. Steendijk P. (1992). Ventricular pressure-volume relations in vivo. *Eur. Heart J.* 13(Suppl. E), 2–6. 10.1093/eurheartj/13.suppl_e.2 1478204

[B3] Backx P. H. Ter Keurs H. E. (1993). Fluorescent properties of rat cardiac trabeculae microinjected with fura-2 salt. *Am. J. Physiol.* 264 H1098–H1110. 8476086 10.1152/ajpheart.1993.264.4.H1098

[B4] Brady A. J. (1967). Length-tension relations in cardiac muscle. *Am. Zool.* 7 603–610. 10.1093/icb/7.3.603 6077380

[B5] Calaghan S. White E. (2004). Activation of Na+–H+ exchange and stretch-activated channels underlies the slow inotropic response to stretch in myocytes and muscle from the rat heart. *J. Physiol.* 559(Pt 1), 205–214. 10.1113/jphysiol.2004.069021 15235080 PMC1665066

[B6] Cuellar A. A. Lloyd C. M. Nielsen P. F. Bullivant D. P. Nickerson D. P. Hunter P. J. (2003). An overview of CellML 1.1, a biological model description language. *Simulation* 79 740–747. 10.1177/0037549703040939

[B7] De Tombe P. P. Little W. C. (1994). Inotropic effects of ejection are myocardial properties. *Am. J. Physiol.* 266 H1202–H1213. 8160824 10.1152/ajpheart.1994.266.3.H1202

[B8] de Tombe P. P. Mateja R. D. Tachampa K. Ait Mou Y. Farman G. P. Irving T. C. (2010). Myofilament length dependent activation. *J, Mol. Cell Cardiol.* 48 851–858. 10.1016/j.yjmcc.2009.12.017 20053351 PMC2854194

[B9] de Tombe P. P. ter Keurs H. E. D. J. (2016). Cardiac muscle mechanics: sarcomere length matters. *J. Mol. Cell Cardiol.* 91 148–150. 10.1016/j.yjmcc.2015.12.006 26678623 PMC5457809

[B10] Downing S. E. Sonnenblick E. H. (1964). Cardiac muscle mechanics and ventricular performance: force and time parameters. *Am. J. Physiol.* 207 705–715. 10.1152/ajplegacy.1964.207.3.705 14220048

[B11] Dowrick J. M. Tran K. Loiselle D. S. Nielsen P. M. F. Taberner A. J. Han J.-C. (2019). The slow force response to stretch: controversy and contradictions. *Acta Physiol.* 226:e13250. 10.1111/apha.13250 30614655

[B12] Frank O. (1899). Die Grundform des arteriellen Pulses. *Z. Biol.* 37 483–526. 10.1016/0022-2828(90)91459-k 2192068

[B13] Garny A. Hunter P. J. (2015). OpenCOR: a modular and interoperable approach to computational biology. *Front. Physiol.* 6:26. 10.3389/fphys.2015.00026 25705192 PMC4319394

[B14] Gibbs C. L. Mommaerts W. F. Ricchiuti N. V. (1967). Energetics of cardiac contractions. *J. Physiol.* 191 25–46. 10.1113/jphysiol.1967.sp008235 6050624 PMC1365437

[B15] Gülch R. W. (1986). “The concept of “end-systolic” pressure-volume and length-tension relations of the heart from a muscle physiologist’s point of view,” in *Controversial Issues in Cardiac Pathophysiology*, ed. Jacob R. (Heidelberg: Steinkopff), 51–57. 10.1007/978-3-662-11374-5_6 3790044

[B16] Han J.-C. Pham T. Taberner A. J. Loiselle D. S. Tran K. (2019). Solving a century-old conundrum underlying cardiac force-length relations. *Am. J. Physiol. Heart Circ. Physiol.* 316 H781–H793. 10.1152/ajpheart.00763.2018 30707611

[B17] Han J.-C. Tran K. Johnston C. M. Nielsen P. M. F. Barrett C. J. Taberner A. J. (2014a). Reduced mechanical efficiency in left-ventricular trabeculae of the spontaneously hypertensive rat. *Physiol. Rep.* 2:e12211. 10.14814/phy2.12211 25413328 PMC4255817

[B18] Han J.-C. Tran K. Nielsen P. M. F. Taberner A. J. Loiselle D. S. (2014b). Streptozotocin-induced diabetes prolongs twitch duration without affecting the energetics of isolated ventricular trabeculae. *Cardiovasc. Diabetol.* 13:79. 10.1186/1475-2840-13-79 24731754 PMC4005834

[B19] Hasenfuss G. Mulieri L. A. Blanchard E. M. Holubarsch C. Leavitt B. J. Ittleman F. (1991). Energetics of isometric force development in control and volume-overload human myocardium. Comparison with animal species. *Circ. Res.* 68 836–846. 10.1161/01.res.68.3.836 1742869

[B20] Hibberd M. G. Jewell B. R. (1982). Calcium- and length-dependent force production in rat ventricular muscle. *J. Physiol.* 329 527–540. 10.1113/jphysiol.1982.sp014317 7143258 PMC1224794

[B21] Hinch R. Greenstein J. L. Tanskanen A. J. Xu L. Winslow R. L. (2004). A simplified local control model of calcium-induced calcium release in cardiac ventricular myocytes. *Biophys. J.* 87 3723–3736. 10.1529/biophysj.104.049973 15465866 PMC1304886

[B22] Hisano R. Cooper G. IV (1987). Correlation of force-length area with oxygen consumption in ferret papillary muscle. *Circ. Res.* 61 318–328. 10.1161/01.res.61.3.318 3621495

[B23] Hofmann P. A. Fuchs F. (1987). Evidence for a force-dependent component of calcium binding to cardiac troponin C. *Am. J. Physiol.* 253 C541–C546. 3499084 10.1152/ajpcell.1987.253.4.C541

[B24] Hongo K. White E. Le Guennec J.-Y. Orchard C. H. (1996). Changes in [Ca2+]i, [Na+]i and Ca2+ current in isolated rat ventricular myocytes following an increase in cell length. *J. Physiol.* 491 609–619. 10.1113/jphysiol.1996.sp021243 8815197 PMC1158804

[B25] Housmans P. R. Lee N. K. Blinks J. R. (1983). Active shortening retards the decline of the intracellular calcium transient in mammalian heart muscle. *Science* 221 159–161. 10.1126/science.6857274 6857274

[B26] Iribe G. Kaneko T. Yamaguchi Y. Naruse K. (2014). Load dependency in force-length relations in isolated single cardiomyocytes. *Prog. Biophys. Mol. Biol.* 115 103–114. 10.1016/j.pbiomolbio.2014.06.005 24976617

[B27] Izakov V. Y. Katsnelson L. B. Blyakhman F. A. Markhasin V. S. Shklyar T. F. (1991). Cooperative effects due to calcium binding by troponin and their consequences for contraction and relaxation of cardiac muscle under various conditions of mechanical loading. *Circ. Res.* 69 1171–1184. 10.1161/01.res.69.5.1171 1934350

[B28] Janssen P. M. de Tombe P. P. (1997). Uncontrolled sarcomere shortening increases intracellular Ca2+ transient in rat cardiac trabeculae. *Am. J. Physiol.* 272 H1892–H1897. 9139976 10.1152/ajpheart.1997.272.4.H1892

[B29] Katsnelson L. B. Izakov V. Y. Markhasin V. S. (1990). Heart muscle: mathematical modelling of the mechanical activity and modelling of mechanochemical uncoupling. *Gen. Physiol. Biophys.* 9 219–243. 2394370

[B30] Kentish J. C. Wrzosek A. (1998). Changes in force and cytosolic Ca2+ concentration after length changes in isolated rat ventricular trabeculae. *J. Physiol.* 506(Pt 2), 431–444. 10.1111/j.1469-7793.1998.431bw.x 9490870 PMC2230716

[B31] Khokhlova A. Konovalov P. Iribe G. Solovyova O. Katsnelson L. (2020). The effects of mechanical preload on transmural differences in mechano-calcium-electric feedback in single cardiomyocytes: experiments and mathematical models. *Front. Physiol.* 11:171. 10.3389/fphys.2020.00171 32256377 PMC7091561

[B32] Kurihara S. Komukai K. (1995). Tension-dependent changes of the intracellular Ca2+ transients in ferret ventricular muscles. *J. Physiol.* 489(Pt 3), 617–625. 10.1113/jphysiol.1995.sp021077 8788928 PMC1156833

[B33] Kurihara S. Saeki Y. Hongo K. Tanaka E. Sudo N. (1990). Effects of length change on intracellular Ca2+ transients in ferret ventricular muscle treated with 2,3-butanedione monoxime (BDM). *Jpn J. Physiol.* 40 915–920. 10.2170/jjphysiol.40.915 2094785

[B34] Lab M. J. Allen D. G. Orchard C. H. (1984). The effects of shortening on myoplasmic calcium concentration and on the action potential in mammalian ventricular muscle. *Circ. Res.* 55 825–829. 10.1161/01.res.55.6.825 6499137

[B35] Landesberg A. (1996). End-systolic pressure-volume relationship and intracellular control of contraction. *Am. J. Physiol.* 270 H338–H349. 8769770 10.1152/ajpheart.1996.270.1.H338

[B36] Landesberg A. Sideman S. (1994). Mechanical regulation of cardiac muscle by coupling calcium kinetics with cross-bridge cycling: a dynamic model. *Am. J. Physiol.* 267 H779–H795. 8067434 10.1152/ajpheart.1994.267.2.H779

[B37] Li K.-L. Ghashghaee N. B. Solaro R. J. Dong W. (2016). Sarcomere length dependent effects on the interaction between cTnC and cTnI in skinned papillary muscle strips. *Arch. Biochem. Biophys.* 601 69–79. 10.1016/j.abb.2016.02.030 26944554 PMC4899114

[B38] Liang B. Chung F. Qu Y. Pavlov D. Gillis T. E. Tikunova S. B. (2008). Familial hypertrophic cardiomyopathy-related cardiac troponin C mutation L29Q affects Ca2+ binding and myofilament contractility. *Physiol. Genomics* 33 257–266. 10.1152/physiolgenomics.00154.2007 18285522

[B39] Markhasin V. S. Solovyova O. Katsnelson L. B. Protsenko Y. Kohl P. Noble D. (2003). Mechano-electric interactions in heterogeneous myocardium: development of fundamental experimental and theoretical models. *Prog. Biophys. Mol. Biol.* 82 207–220. 10.1016/s0079-6107(03)00017-8 12732280

[B40] Nickerson D. Buist M. (2008). Practical application of CellML 1.1: the integration of new mechanisms into a human ventricular myocyte model. *Prog. Biophys. Mol. Biol.* 98 38–51. 10.1016/j.pbiomolbio.2008.05.006 18606438

[B41] Niederer S. A. Hunter P. J. Smith N. P. (2006). A quantitative analysis of cardiac myocyte relaxation: a simulation study. *Biophys. J.* 90 1697–1722. 10.1529/biophysj.105.069534 16339881 PMC1367320

[B42] Niederer S. A. Smith N. P. (2007). A mathematical model of the slow force response to stretch in rat ventricular myocytes. *Biophys. J.* 92 4030–4044. 10.1529/biophysj.106.095463 17369410 PMC1868992

[B43] Palmer S. Kentish J. C. (1998). Roles of Ca2+ and crossbridge kinetics in determining the maximum rates of Ca2+ activation and relaxation in rat and guinea pig skinned trabeculae. *Circ. Res.* 83 179–186. 10.1161/01.res.83.2.179 9686757

[B44] Parikh S. S. Zou S. Z. Tung L. (1993). Contraction and relaxation of isolated cardiac myocytes of the frog under varying mechanical loads. *Circ. Res.* 72 297–311. 10.1161/01.res.72.2.297 8418985

[B45] Parvatiyar M. S. Landstrom A. P. Figueiredo-Freitas C. Potter J. D. Ackerman M. J. Pinto J. R. (2012). A mutation in TNNC1-encoded cardiac troponin C, TNNC1-A31S, predisposes to hypertrophic cardiomyopathy and ventricular fibrillation. *J. Biol. Chem.* 287 31845–31855. 10.1074/jbc.M112.377713 22815480 PMC3442518

[B46] Parvatiyar M. S. Pinto J. R. Dweck D. Potter J. D. (2010). Cardiac troponin mutations and restrictive cardiomyopathy. *J. Biomed. Biotechnol.* 2010:350706. 10.1155/2010/350706 20617149 PMC2896668

[B47] Pinto J. R. Parvatiyar M. S. Jones M. A. Liang J. Ackerman M. J. Potter J. D. (2009). A functional and structural study of troponin C mutations related to hypertrophic cardiomyopathy. *J. Biol. Chem.* 284 19090–19100. 10.1074/jbc.M109.007021 19439414 PMC2707221

[B48] Pironet A. Desaive T. Kosta S. Lucas A. Paeme S. Collet A. (2013). A multi-scale cardiovascular system model can account for the load-dependence of the end-systolic pressure-volume relationship. *Biomed. Eng. Online* 12:8. 10.1186/1475-925X-12-8 23363818 PMC3610305

[B49] Rice J. J. de Tombe P. P. (2004). Approaches to modeling crossbridges and calcium-dependent activation in cardiac muscle. *Prog. Biophys. Mol. Biol.* 85 179–195. 10.1016/j.pbiomolbio.2004.01.011 15142743

[B50] Rice J. J. Wang F. Bers D. M. de Tombe P. P. (2008). Approximate model of cooperative activation and crossbridge cycling in cardiac muscle using ordinary differential equations. *Biophys. J.* 95 2368–2390. 10.1529/biophysj.107.119487 18234826 PMC2517033

[B51] Rogers J. M. McCulloch A. D. (1994). A collocation–Galerkin finite element model of cardiac action potential propagation. *IEEE Trans. Biomed. Eng.* 41 743–757. 10.1109/10.310090 7927397

[B52] Shimizu J. Todaka K. Burkhoff D. (2002). Load dependence of ventricular performance explained by model of calcium-myofilament interactions. *Am. J. Physiol. Heart Circ. Physiol.* 282 H1081–H1091. 11834507 10.1152/ajpheart.00498.2001

[B53] Solaro R. J. Kobayashi T. (2011). Protein phosphorylation and signal transduction in cardiac thin filaments. *J. Biol. Chem.* 286 9935–9940. 10.1074/jbc.r110.197731 21257760 PMC3060547

[B54] Sørhus V. Sys S. U. Natåns A. Demolder M. J. Angelsen B. A. (2000). Controlled auxotonic twitch in papillary muscle: a new computer-based control approach. *Comput. Biomed. Res.* 33 398–415. 10.1006/cbmr.2000.1551 11150234

[B55] Suga H. Hayashi T. Shirahata M. Suehiro S. Hisano R. (1981). Regression of cardiac oxygen consumption on ventricular pressure-volume area in dog. *Am. J. Physiol.* 240 H320–H325. 7212076 10.1152/ajpheart.1981.240.3.H320

[B56] Suga H. Sagawa K. (1974). Instantaneous pressure-volume relationships and their ratio in the excised, supported canine left ventricle. *Circ. Res.* 35 117–126. 10.1161/01.res.35.1.117 4841253

[B57] Syomin F. A. Tsaturyan A. K. (2017). A simple model of cardiac muscle for multiscale simulation: passive mechanics, crossbridge kinetics and calcium regulation. *J. Theor. Biol.* 420 105–116. 10.1016/j.jtbi.2017.02.021 28223172

[B58] Taberner A. J. Han J.-C. Loiselle D. S. Nielsen P. M. F. Nielsen P. M. F. (2011). An innovative work-loop calorimeter for in vitro measurement of the mechanics and energetics of working cardiac trabeculae. *J. Appl. Physiol.* 111 1798–1803. 10.1152/japplphysiol.00752.2011 21903883

[B59] Taylor R. R. Covell J. W. Ross J. Jr. (1969). Volume-tension diagrams of ejecting and isovolumic contractions in left ventricle. *Am. J. Physiol.* 216 1097–1102. 10.1152/ajplegacy.1969.216.5.1097 5797292

[B60] ter Keurs H. E. Rijnsburger W. H. van Heuningen R. Nagelsmit M. J. (1980). Tension development and sarcomere length in rat cardiac trabeculae. Evidence of length-dependent activation. *Circ. Res.* 46 703–714. 10.1161/01.res.46.5.703 7363419

[B61] Terkildsen J. R. Niederer S. Crampin E. J. Hunter P. Smith N. P. (2008). Using Physiome standards to couple cellular functions for rat cardiac excitation-contraction. *Exp. Physiol.* 93 919–929. 10.1113/expphysiol.2007.041871 18344258

[B62] Tran K. Han J.-C. Crampin E. J. Taberner A. J. Loiselle D. S. (2017). Experimental and modelling evidence of shortening heat in cardiac muscle. *J. Physiol.* 595 6313–6326. 10.1113/JP274680 28771742 PMC5621496

[B63] Tran K. Han J.-C. Taberner A. J. Barrett C. J. Crampin E. J. Loiselle D. S. (2016). Myocardial energetics is not compromised during compensated hypertrophy in the Dahl salt-sensitive rat model of hypertension. *Am. J. Physiol. Heart Circ. Physiol.* 311 H563–H571. 27402668 10.1152/ajpheart.00396.2016 PMC5142187

[B64] Tran K. Smith N. P. Loiselle D. S. Crampin E. J. (2010). A metabolite-sensitive, thermodynamically constrained model of cardiac cross-bridge cycling: implications for force development during ischemia. *Biophys. J.* 98 267–276. 10.1016/j.bpj.2009.10.011 20338848 PMC2808479

[B65] Tran K. Taberner A. J. Loiselle D. S. Han J.-C. (2020). Energetics equivalent of the cardiac force-length end-systolic zone: implications for contractility and economy of contraction. *Front. Physiol.* 10:1633. 10.3389/fphys.2019.01633 32038302 PMC6985585

[B66] Weber K. T. Janicki J. S. Hefner L. L. (1976). Left ventricular force-length relations of isovolumic and ejecting contractions. *Am. J. Physiol.* 231 337–343. 10.1152/ajplegacy.1976.231.2.337 961884

[B67] White E. Boyett M. R. Orchard C. H. (1995). The effect of mechanical loading and changes of length on single guinea-pig ventricular myocytes. *J. Physiol.* 482 93–107. 10.1113/jphysiol.1995.sp020502 7730993 PMC1157756

[B68] Yasuda S.-I. Sugiura S. Yamashita H. Nishimura S. Saeki Y. Momomura S.-I. (2003). Unloaded shortening increases peak of Ca2+ transients but accelerates their decay in rat single cardiac myocytes. *Am. J. Physiol. Heart Circ. Physiol.* 285 H470–H475. 12714336 10.1152/ajpheart.00012.2003

