# Supplementary material for: Insights From Computational Modeling Into the Contribution of Mechano-Calcium Feedback on the Cardiac End-Systolic Force-Length Relationship
Source: Front Physiol. 2020 May 29;11:587. doi: 10.3389/fphys.2020.00587 (PMC7273927; doi:10.3389/fphys.2020.00587)
Supplement: Supplementary file 1 [file Data_Sheet_1.PDF]

Supplementary Material

1 Supplementary Figures

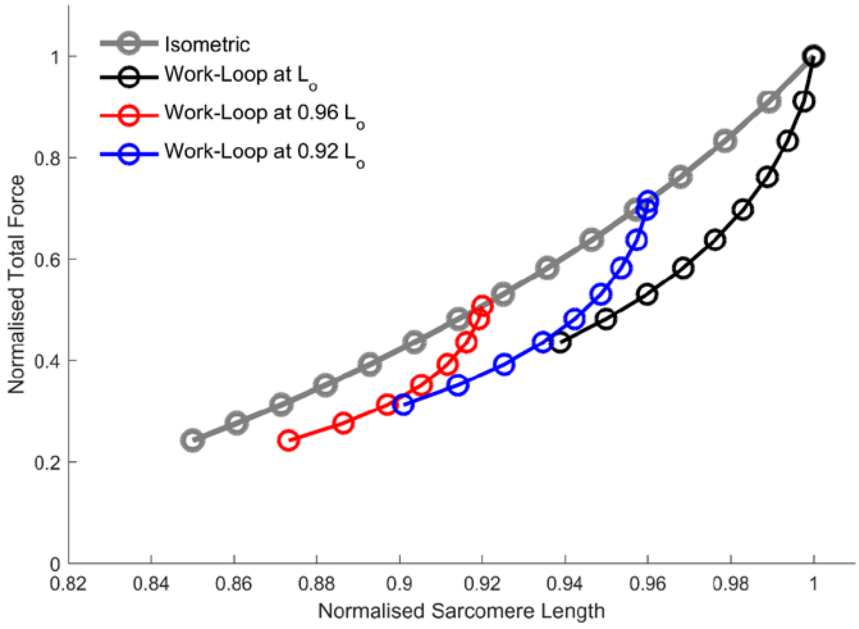

Supplementary Figure 1. Simulation of isometric and work-loop end-systolic force-length relations (ESFLR) at different initial sarcomere lengths. L_o is the sarcomere length that produces maximum active force. As the initial sarcomere length is reduced from L_o to 0.96 L_o and 0.92 L_o, the resulting work-loop ESFLR form a family of curves that locate progressively closer to the isometric ESFLR. These model-predicted work-loop ESFLR are consistent with recent experimental data quantifying the preload dependence of these relations in rat trabeculae (Han et al. 2019).

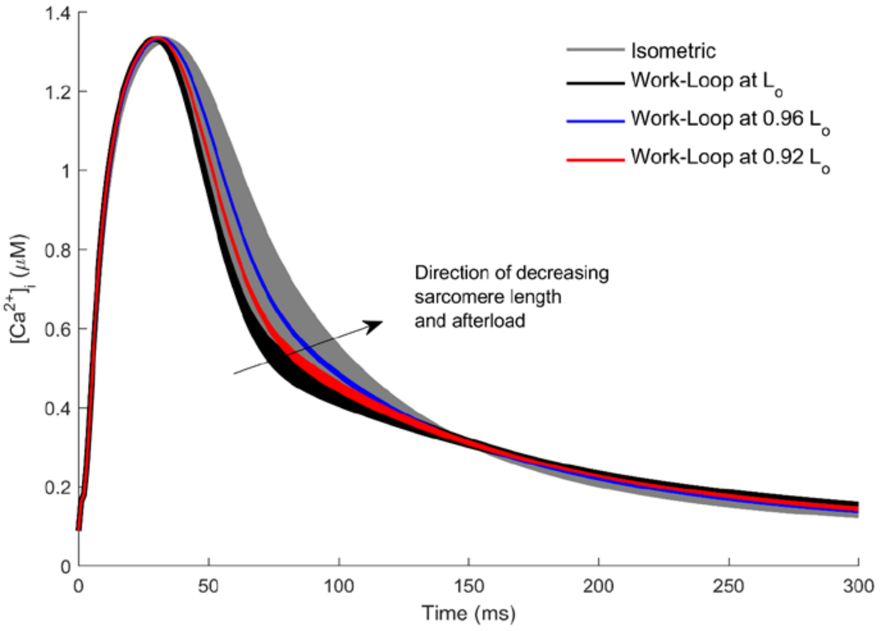

Supplementary Figure 2. Simulation of dynamic Ca^{2+} transients associated with isometric and work-loop ESFLRs presented in Supplementary Figure 1. Each of the coloured shaded regions contain a series of Ca^{2+} transients elicited by either changing sarcomere length in the isometric case, or changing afterload in the work-loop case. As the initial sarcomere length for work-loop contractions in decreased from L_o to 0.96 L_o and 0.92 L_o, the reduction in active force leads to a widening of the Ca^{2+} transient.

2 References

Han J-C, Pham T, Taberner AJ, Loiselle DS & Tran K (2019). Solving a century-old conundrum underlying cardiac force-length relations. *Am J Physiol Heart Circ Physiol* **316,** H781–H793.
